# Pedicle Screw-Based Posterior Dynamic Stabilization: Literature Review

**DOI:** 10.1155/2012/424268

**Published:** 2012-11-28

**Authors:** Dilip K. Sengupta, Harry N. Herkowitz

**Affiliations:** ^1^Department of Orthopedics, Dartmouth-Hitchcock Medical Center, 1 Medical Center Drive, Lebanon, NH 03756, USA; ^2^Department of Orthopaedic Surgery, Beaumont Hospital, Oakland University William Beaumont School of Medicine, 3535 West 13 Mile Road, Royal Oak, MI 48073, USA

## Abstract

Posterior dynamic stabilization (PDS) indicates motion preservation devices that are aimed for surgical treatment of activity related mechanical low back pain. A large number of such devices have been introduced during the last 2 decades, without biomechanical design rationale, or clinical evidence of efficacy to address back pain. Implant failure is the commonest complication, which has resulted in withdrawal of some of the PDS devices from the market. In this paper the authors presented the current understanding of clinical instability of lumbar motions segment, proposed a classification, and described the clinical experience of the pedicle screw-based posterior dynamic stabilization devices.

## 1. Introduction

The mechanism and surgical treatment of axial mechanical back pain remain controversial. The concept of instability as a cause of activity related, mechanical back pain is not well defined, and poorly understood. Yet, most clinicians like to believe that some form of instability, be it abnormal motion, or abnormal load transmission, is a crucial factor in the pathophysiology of mechanical back pain secondary to disc and/or facet degeneration [1]. Spinal fusion had been the cornerstone of surgical treatment for back pain during the last three decades [2]. While fusion works in the majority of the patients, in many cases persistent back pain despite a solid fusion continues to haunt the surgeons and patients [3]. Besides, accelerated degeneration of the adjacent segment after initial clinical success with fusion surgery is fairly common. The concept of dynamic stabilization was developed out of failure of fusion to deliver the desirable clinical result. Dynamic stabilization indicates control of motion and/or load bearing of the motion segment, to address instability and the resultant back pain.

## 2. Spinal Instability in Degenerative Disorder as a Cause of Back Pain 

Instability is commonly understood as loss of stiffness or an increased motion to a given load, as originally defined by Pope and Panjabi in 1985 [4]. In presence of an abnormal motion like horizontal translation on flexion-extension radiographs, especially in the setting of spondylolisthesis, a clinical instability is considered to be present [5]. By this standard, however, relatively few patients with low back pain have overt subjective or objective evidence of instability. Radiological studies using open MRI in flexion and extension have shown that segmental motion either does not change significantly with the disc degeneration [6–8] or may in fact decrease, except during early stages of disc degeneration [9].

More recently, Panjabi redefined spinal instability as an abnormal motion often accompanied by an increased neutral zone (NZ) motion caused by ligament laxity, even when the ROM is diminished [10]. Panjabi uses the analogy of a marble rolling on a soup bowl [1]. 

Mulholland and Sengupta [11] hypothesize that spinal instability does not mean “increased motion” as commonly misunderstood [4, 12], but it indicates abnormal load distribution across the vertebral end plate. In normal hydrated disc, homogeneous gel of collagen and proteoglycan act like a fluid-filled bag, that allows uniform load distribution across the vertebral endplates [11]. In a degenerated collapsed disc, the hydration of the nucleus is lost, and load transmission across the vertebral endplate becomes irregular. Most of the load is transmitted directly from bone to bone towards the periphery of the endplate [13]. The annulus and endplate cartilage are fragmented and may act like a “stone in the shoe” which may cause a high point loading and pain, a concept proposed by Mulholland and Sengupta [11]. This hypothesis of abnormal loading as the primary cause of mechanical back pain was supported by a close association of abnormal disc pressure profiles to positive discography with pain provocation [14, 15]. The Modic changes associated with disc degeneration, as seen in the MRI scan, change over time form oedema followed by sclerosis and may represent the effect of reaction of the cancellous bone to abnormal stress or load-bearing. This may be an indirect evidence in support of abnormal load bearing theory proposed by Mulholland.

Abnormal motion and abnormal load distribution may be interrelated and there may be no real conflict between the motion (Panjabi) and load distribution (Mulholland) theories of spinal instability. An abnormal motion may lead to an abnormal load distribution leading to pain. Conversely, if an abnormal motion does not cause abnormal load distribution, it may not be associated with pain production. The abnormal load concept may also help explain the lack of correlation between degrees of disc degeneration and back pain. The magnitude of abnormal load transmission may vary highly between individuals with similar degree of disc degeneration, and even in the same individual from time to time, causing acute painful episodes in the setting of chronic low back pain. With advanced degeneration, complete collapse of the disc may once again distribute loads more evenly, resulting in a degree of spontaneous relief of pain with advancing age [16].

## 3. Definitions

 “Motion preservation devices” in the treatment of degenerative low back pain may be classified as prosthetic devices, and dynamic stabilization devices. Total disc replacement (TDR), nucleus replacement devices (e.g., PDN, Raymedica, Inc., Minneapolis, MN), and facet replacement devices like ACADIA (Globus Medical, Inc., Audobon, PA) and TOPS (Premia Spine, Ltd., Herzeila, Israel) are prosthetic devices, as they replace anatomical structure and function in the lumbar motion segment. In contrast, dynamic stabilization devices work in conjunction with the motion segment, without replacing any anatomical part of it.

The posterior dynamic stabilization (PDS) devices are posterior pedicle screw-based flexible devices, which intend to control motion and/or load bearing of the motion segment, to address instability and the resultant back pain [1]. 

The other major group of dynamic stabilization devices comprises Interspinous Process Distraction devices (IPD), which are essentially floating devices that do not require any bony anchorage like pedicle screw insertion. *Semirigid fixation* is the term used to describe the devices, intended for achieving solid fusion without the stress shielding effect of conventional rigid fixation. These are often flexible metallic devices of various designs, as opposed to conventional fusion rods, which offer no mobility at the instrumented segment. Typical devices in this category are Isobar TTL (Scient'x, Inc., West Chester, PA) and Accuflex (Globus Medical, Inc., Audobon, PA), and so forth [1]. The true pedicle screw-based posterior dynamic stabilization devices like Dynesys and Transition, are introduced in the US market as a fusion device under 510 (k) approval from FDA. This has lead to frequent use of these devices as fusion devices. The argument in favor of using dynamic stabilization devices for semirigid fixation over rigid fixation to achieve fusion is that, the fusion mass may be more robust, being free from stress shielding. There has been no clinical study to establish this concept. The current paper however focuses the discussion on the regular use of these devices as true posterior dynamic stabilization without fusion and without the use of bone graft at the index level.

## 4. Design Rationale for the Posterior Dynamic Stabilization Devices

During the last decade, a large number of PDS devices were introduced, with very little understanding of their mechanism of action. The common factor of all these devices is restriction of “some motion” and some degree of load sharing by the device with the motions segment. Thus, in the short term, any device may be effective to reduce back pain to some extent. For long-term survival, the load sharing and motion control by the device should be complementary to the kinematics of the motion segment, through the range of motion. The device may end up in fatigue failure, if there is a conflict in kinematics between the device and the motion segment. For example, if the device becomes total-load bearing structure at certain phase of motion, which is not uncommon towards the end of extension in case of posterior dynamic stabilization devices, the device may fail eventually. This has been explained further in the following sections.

Biomechanical goals for posterior dynamic stabilization are as follows:

1. motion preservation,
2. load transmission.

The goal is to preserve as much of normal motion as possible, but to limit any abnormal motion. Some degree of loss of motion is inevitable with application of any PDS device. 

Normally, it is unlikely that a dynamic stabilization device will increase the ROM of a degenerated segment. On rare occasions, however, it may be expected that the device may restore the disc height from collapsed state by distraction and eventually may increase the ROM to some degree. When the ROM is abnormal qualitatively or quantitatively, for example, following laminectomy or discectomy, the goal of posterior dynamic stabilization should be to restore a normal range and quality of motion. Regardless of the magnitude of motion preservation, the device must ensure normal load distribution across the endplate, throughout the range of motion, in order to achieve painless motion. 

The challenges of the PDS devices are as follows:

1. fatigue failure,
2. pedicle-to-pedicle distance excursion.

Unlike fusion devices, the PDS device has to survive fatigue failure for an indefinite period. Since the PDS devices need to work in conjunction with the normal anatomical structures of the motion segment, it is essential that there is no conflict in kinematic and kinetic properties of the motion segment and the PDS device. For load transmission the PDS device should work as a “load-sharing device,” and should not become a load-bearing device at any stage, which may lead to fatigue failure. For controlling ROM, the device should allow pedicle-to-pedicle excursion in both opposing directions in three planes of motion. The clue to the device failure (screw loosening or breakage), as has been discussed in the following sections, may be hidden in the fact that device may act as an extension stop, denying pedicle excursion, and behaves like a total load-bearing device in extension [1].

The other design related factors are as follows:

- safe and easy salvage—conversion to fusion in case of failure,
- ease of implantation—top-loading screws,
- compatibility with Minimally Invasive Procedure,
- restoration of lordosis,
- biomaterial—metallic versus non-metallic.

## 5. Dynamic Stabilization Devices

The classification of dynamic stabilization devices is a moving target; new devices are being introduced, and some devices are being constantly withdrawn. As defined earlier, only the PDS devices are included in the classification presented in Table 1. The IPD devices, semirigid fixation devices, and prosthetic devices, including facet replacement devices, were excluded.

The primary indication for posterior dynamic stabilization is to address activity related mechanical axial back pain. Another common indication is to prevent progression of degeneration of segment adjacent to fusion which shows early signs of degeneration, often referred to as “topping off.” Prevention of iatrogenic spinal instability following decompression and to supplement Total Lumbar Disc Replacement with posterior stabilization are considered secondary indications. The indications are summarized in Table 2.

Unfortunately, many PDS devices have been introduced to address secondary indications, without establishing their efficacy to address any of the primary indications. Once that is established, application in conjunction with decompression procedures could be justified. Dynamic stabilization to supplement total disc replacement is still at an experimental stage and may be considered as a future indication [1].

## 6. Clinical Experience with Posterior Dynamic Stabilization

**Graf Ligament**

Henry Graf introduced the earliest PDS device in the treatment of low back pain in 1992 [17]. This may be considered as the *first generation PDS device*. This is a very simple device, consisting of braided polypropylene circular bands, which is looped around the pedicle screw heads under tension. Essentially, the device locks the facet joints under compression, presumably preventing any abnormal and pain movement, the so-called instability.

Henry Graf never presented any peer reviewed article on the design rationale, or mechanism of action of Graf ligament. The exact mechanism of action of the Graf ligaments therefore remains a matter of educated guess rather than established on a sound biomechanical basis. The apparent clinical success may project Graf ligament as an attractive surgical option, particularly in young subjects with intractable back pain secondary to multilevel disc degeneration, where fusion is a difficult choice. Unfortunately, as a result of the compression applied to the screws, there is a high incidence of radicular symptoms secondary to either disc herniation or foraminal narrowing [18, 19]. The compressive force may also have a deleterious effect on the facet joint and may lead to back pain.

Short-term clinical outcome (up to 2 years) with Graf ligament is reported to be comparable to conventional fusion [19]. Long-term outcome has conflicting reports. Gardner and Pande [20] and Markwalder and Wenger [21] reported reasonably good result with Graf ligament even at 5–10-year followup. On the other hand, Hadlow et al. [18] reviewed their outcome with Graf ligament stabilization in 83 consecutive patients, and reported a worse outcome at 1 year and a significantly higher revision rate at 2 years, compared to instrumented fusion. The Graf ligament is still being used in a few centers in both Europe and Asia, but its use has declined [20, 22].

**Dynesys**

The most extensively used PDS device around the world is Dynesys (Zimmer Spine Inc., Warsaw, IN) [23, 24]. The design rationale is based on improvement over Graf ligament, preventing compression between the screws by introduction of a polythene spacer. This device may therefore be called *a second generation PDS device.* The plastic cylinder (sulene-polycarbonate urethane (PCU)) is placed around the cord to apply a distraction force between the pedicle screws, and thereby unloading the facet joints, which addresses a disadvantage of Graf ligament.

The biomechanical effect of Dynesys on the range of motion as seen in cadaver experiments in vitro is diametrically opposite to its in vivo effect after implantation in patients. In cadaver spine, Dynesys holds the motion segment in near full flexion and permits minimal further flexion [25]. It can still allow significant extension by an abnormal distraction of the disc space, with the plastic cylinder acting like a fulcrum. This is evidenced by an abnormal negative disc pressure during extension [26]. Conversely, in vivo Dynesys limits extension more than flexion [27], The device acts like an extension stop and becomes a totally load-bearing structure in extension. This may explain why screw loosening or breakage had been so rare with Graf ligament, but fairly common with Dynesys, as high as 17% in some clinical series [23, 28].

In the initial clinical report, presented by the inventors (Stoll et al.), Dynesys produced clinical success comparable to fusion [24]. However, since over 60% of their cases had spinal stenosis and concomitant decompression, it is difficult to evaluate whether the good outcome was primarily due to Dynesys or the decompression. The clinical success rate drops to only 39% after stand-alone stabilization with Dynesys without accompanying decompression [23, 29].

Dynesys was introduced in the United States in 2004 with FDA approval under 510 k as a fusion device [30]. Its sporadic use as a nonfusion device represents an off-label use. An FDA controlled investigations device exemption (IDE) clinical trial was completed in 2009, comparing Dynesys, as a stand-alone dynamic stabilization device against instrumented fusion. The preliminary report showed promising outcome [30].

Unfortunately, FDA executive panel did not approves use of Dynesys as a stand-alone device for nonfusion stabilization on the basis of the noninferiority study for various shortcomings of the study. The detail of the FDA Executive Summary can be found on its web site [31].

A comprehensive review of the literature on the Dynesys system was conducted by the National Institute for Clinical Excellence (NICE) of the National Health Service (NHS) in the United Kingdom in 2009. They reviewed outcome studies that included a total of 743 patients from 4 nonrandomized comparative studies and 3 case series, published between 2001 and 2007. This review concluded that the use of Dynesys is both safe and efficacious as a dynamic stabilization technique for some patients with intractable lumbar pain [32].

**Transition**

Stabilization System (Globus Medical Inc., Audubon, PA) was evolved from Dynesys, addressing its several design limitations. It may therefore be considered as a *third generation PDS device*. It consists of a cylindrical polycarbonate urethane (PCU) spacer around a polyethylene terephthalate (PET) cord similar to Dynesys. There are three major design improvements in Transition system compared to Dynesys: (i) use of regular top-loading pedicle screw, (ii) active restoration of lordosis with instrumentation, and (iii) permiting increased pedicle-to-pedicle distance excursion by use of an additional bumper. The other major design improvement over Dynesys is that the system comes preassembled, avoiding the need for assembly from components and tensioning of the cord directly onto the spine at surgery. The use of regular, top-loading pedicle screws simplifies implantation of the system, and conversion to fusion as a salvage procedure when needed. While Dynesys may potentially cause loss of lordosis, this system creates an active lordosis of the instrumented segment. Finally, Transition system is adapted for application adjacent to a rigid instrumented fusion segment, because it uses regular pedicle screws for the rigid rod as well as flexible component. Although this device has made several design improvements compared to Dynesys, its advantages in the clinical practice remain to be established. Transition device has only been recently approved by the FDA under 510 K as a fusion device, but no clinical outcome has been reported in the peer reviewed literature yet [1].

**The BioFlex**

The BioFlex (BioSpine Corporation, Seoul, Korea) [33] consists of a Nitinol coil spring made of 4 mm diameter wires, which is applied between the pedicle screws. Nitinol provides increased flexibility. The device was developed in Seoul, Korea. This device has been used most commonly in conjunction with interbody cages to achieve fusion, although it has also been used as a stand-alone nonfusion device [34]. Recently, a titanium version of the device has been approved by the FDA under 510 (k) as a fusion device, but no clinical use in the US has been reported yet.

**Stabilimax NZ**

Stabilimax NZ (Rachiotek, Wellesley, MA) [35], was developed by Panjabi. This system consists of a dual core spring device, designed to apply soft resistance against both compression and distraction. The design rationale is to limit the NZ motion but leaving the elastic zone unaffected as much as possible. This device started an FDA controlled IDE clinical trial to assess whether the Stabilimax NZ has equivalent safety and efficacy compared to fusion in patients receiving decompression surgery for the treatment of clinically symptomatic spinal stenosis at one or two contiguous vertebral levels from L1-S1 [36]. Due to initial screw failure the manufacturer AST voluntarily suspended enrollment in August 2008; following appropriate device modification the trial resumed enrolment in 2009.

**Cosmic**

Posterior Dynamic System (Ulrich GmbH & Co, Ulm, Germany) [37] has a unique design; unlike conventional PDS devices, the rods connecting the pedicle screws are rigid. The pedicle screws have hinged head, which permits motion. This is described as a combination of rigid rod and dynamic pedicle screws, which produces posterior dynamic transpedicular stabilization (PDTS). No biomechanical data is available in the peer reviewed literature. In a prospective clinical study with minimum 2-year followup Kaner et al. [38] reported an equivalent clinical outcome with Cosmic stabilization system (*n* = 26) versus rigid fusion (*n* = 20) in degenerative spondylolisthesis. In another clinical outcome study the same group of authors reported that Cosmic stabilization was found safe and effective in 40 patients with recurrent disc herniation treated with microdiscectomy and posterior dynamic transpedicular stabilisation at minimum 2-year followup [39].

The Hybrid Devices incorporate a combination of metallic rod connected to a flexible segment, which consists of a non-metallic bumper. The design rationale is to allow shock absorption, as well as some degree of pedicle-to-pedicle excursion. These devices can be used with a regular top-loading pedicle screw like a regular rigid fusion rod, and therefore can be used for “topping off” an adjacent segment to rigid fusion.

**The CD Horizon Agile**

The CD Horizon Agile (Medtronic, Memphis, TN) system incorporates a plastic cylindrical bumper at the end of a fusion rod, held by a metallic cable in its center. This system was launched in 2007, for single-level dynamic stabilization or as a so-called topping-off hybrid construct adjacent and superior to fusion. This was a multicenter, observational study for both configurations to determine the safety and efficacy of this new implant. The study was terminated in December 2007 because of the recall of the implant due to high failure rates. One of the study sites recently reported outcome of 40 patients (18 single level stabilization, and 22 “topping off” adjacent to fusion) enrolled at that center, and explored which radiographic parameters are linked to failure of this device. 37/40 patients completed 2-year followup, of which 10 (27%) had implant failure. The authors found two important factors predictive of implant failures are greater disc height and “Implant translation”. The authors concluded that implant translation was associated with high failure rate, which is due to insufficient resistance to shear forces by the implant [40].

**N-Flex**

N-Flex (Synthes Spine Inc., West Chester, PA, a J&J Company) was designed to accommodate physiologic motion via a bumper element that permits elongation and angulation during flexion and allows compression during extension. The system is semirigid and composed of a titanium rod with one end containing a composite titanium-polycarbonate urethane sleeve positioned over the titanium core. Although the device may permit compression and elongation, its ability to permit anteroposterior translation remains a concern. The device has been used clinically since fall of 2006 [41]. In a biomechanical study in cadaver spine (*n* = 7), Mageswaran et al. reported that the system displayed stability characteristics similar to a solid, all-metal construct. The system essentially transformed a 1-level lumbar fusion into a 2-level lumbar fusion, with exponential transfer of motion to the fewer remaining discs [42].

## 7. Summary

Pedicle screw-based posterior dynamic stabilization evolved from failure of fusion to address mechanical back pain due to spinal instability. The current understanding of spinal instability is abnormal quality of motion, leading to uneven load transmission. The primary biomechanical goals of PDS devices are to preserve motion as much as possible, but to prevent any abnormal motion, and to unload the disc and facet joints by load sharing. Survival against fatigue failure is the biggest challenge for PDS device because of the need for continued motion for an indefinite period. The key to this survival is uniform load sharing throughout the range of motion. 

Most of the new PDS devices are introduced in the US market with FDA approval under 510 (k) as a fusion device. The clinical use for dynamic stabilization without an attempt of fusion becomes an “off-label” use. Dynamic stabilization without fusion in the surgical treatment of activity related mechanical back pain raised a great deal of enthusiasm, theoretical promises, and many expectations. Implant failures resulted in some of these devices being withdrawn from the market. The initial enthusiasm of developing newer PDS devices has been dampened with the failure of Dynesys to obtain FDA approval for use as a stand-alone stabilization device. 

Fusion still remains the method of choice for advanced disc/facet degeneration and gross instability. However, disc degeneration in multiple segments, particularly in young patients with concerns about adjacent segment disease following fusion, will likely constitute the main indication for posterior dynamic stabilization. Further demand for PDS devices is raised for salvage of failed TDR or nucleus replacement. Total joint replacement of a motion segment will require TDR and PDS devices together, so that they can act complementary to each other, without any conflict in their kinetic and kinematic properties. Future application of PDS devices may include temporary mechanical support for pharmacological and/or genetic treatment aiming for repair or regeneration of the disc. 

## Figures and Tables

**Table 1:** Classification of the pedicle screw-based posterior dynamic stabilization devices.

(A) Nonmetallic devices	
(i) Graf Ligament (SEM Co., Montrouge, France)	
(ii) Dynesys (Zimmer Spine, Inc., Warsaw, IN)	
(iii) Transition (Globus Medical, Inc., Audubon, PA)	
(B) Metallic devices	
(i) BioFlex (Bio-Spine Corp., Seoul, Korea)	
(ii) Stabilimax NZ (Applied Spine Technologies, Inc., New Haven, CT)	
(iii) Cosmic Posterior Dynamic System (Ulrich GmbH & Co, Ulm, Germany)	
(C) Hybrid devices	
(a) Hybrid devices (metallic component with plastic bumper)	
(i) CD Horizon Agile (Medtronic Sofamor Danek, Memphis, TN)	
(ii) NFlex (Synthes Spine, Inc., West Chester, PA, a J&J Company)	

**Table 2:** Indications for posterior dynamic stabilization.

(i) Primary indication: treatment of spinal instability	
(a) Activity related mechanical back pain secondary to disc/facet degeneration (disc degeneration, facet degeneration, degenerative spondylolisthesis)	
(b) Prevent accelerated degeneration to an adjacent motion segment with signs of early degeneration	
(ii) Secondary indications: prevention of spinal instability	
(a) Laminectomy	
(b) To achieve solid fusion—free from stress shielding	
(iii) Future indications:	
(a) Supplement TDR to achieve total joint replacement	
